# „Schenkelhalsklingen-Cut-in“ nach Osteosynthese einer pertrochantären Femurfraktur mittels TFNA©

**DOI:** 10.1007/s00113-022-01178-9

**Published:** 2022-04-27

**Authors:** Mathias Reimond, Thomas Gross

**Affiliations:** 1 Orthopädische Klinik, Traumatologie, Kantonsspital Bruderholz, 4101 Bruderholz, Schweiz; 2 Universität Basel, Basel, Schweiz

**Keywords:** Cut-in, Spiralklinge, Osteosynthese, Trochantäre Femurfraktur, Fixationsversagen, Cut-in, Internal fixation, Trochanteric hip fracture, Helical blade, Fixation failure

## Abstract

Ein 96-jähriger Patient erlitt nach der TFNA**©**-Osteosynthese einer pertrochantären Femurfraktur trotz korrekter Reposition und Fixation einen vollständigen „cut-in“, d. h. eine Medialisierung der gesamten Schenkelhalsklinge im Nagel ins Hüftgelenk. Vor dem Hintergrund der Implantatentwicklung und anhand der aktuellen Literatur wird diese häufiger werdende Komplikationsart beschrieben bzw. vom Fixationsversagen des „cut-out“ unterschieden. Bisherige Erklärungsversuche und notwendige Forschungsansätze werden aufgezeigt und abschließend ein pragmatisches Vorgehen im Alltag dargelegt.

## Abstract (en)

Following fixation of a trochanteric femoral fracture with a TFNA© and despite correct fracture reduction and fixation, a 96-year-old patient suffered a complete cut-in, i.e. a medialization of the entire femoral neck blade through the nail into the hip joint. Against the background of implant development and current literature, this increasingly frequent type of complication is described and distinguished from other fixation failures such as the cut-out. Attempts to explain this phenomenon as well as research options are presented and a pragmatic approach to avoid this problem is outlined.

## Fallbeschreibung

### Anamnese

Der 96-jährige Herr A. stolperte zu Hause und stürzte auf die rechte Körperseite, mit Schmerzen im Bereich der rechten Hüfte. An Nebendiagnosen u. a. Marcoumarisierung nach Lungenembolie, anamnestisch keine Osteoporose bekannt bei St. n. Hüfttotalprothese links vor Jahren.

### Klinischer Befund

Klinisch fand sich ein verkürztes, außenrotiertes Bein rechts bei unauffälliger Sensibilität und Durchblutung des rechten Beines.

### Diagnostik

**Figure 1:**
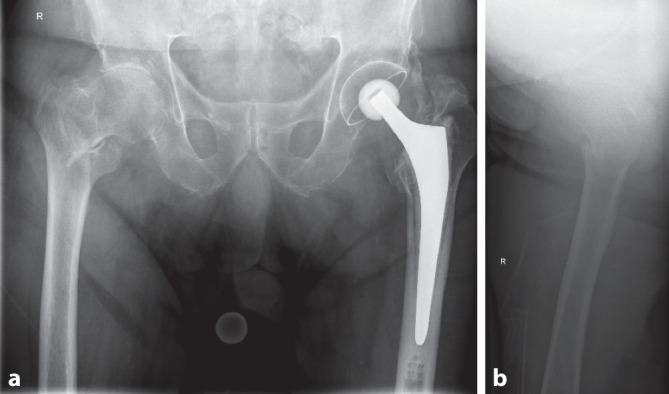

Das Röntgen (Rx) des Beckens bzw. der rechten Hüfte zeigte eine pertrochantäre Femurfraktur (AO-Typ A1.2; Abb. 1). Die radiologisch dokumentierte Hüftarthrose (bei St. n. Totalhüftprothesenversorgung auf der Gegenseite vor Jahren) war anamnestisch für den Patienten nicht limitierend.

### Therapie und Verlauf

**Figure 2:**
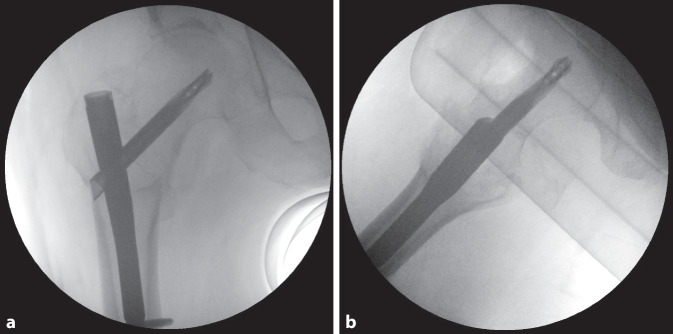

Es erfolgte die osteosynthetische Versorgung mittels dem an unserer Klinik zu jenem Zeitpunkt für diese Frakturen standardmäßig verwendeten proximalen Femurnagel, dem TFNA© (Fa. DePuy/Synthes, Zuchwil, Schweiz). Intraoperativ problemloser Ablauf mit annähernd anatomischer, geschlossener Reposition auf dem Extensionstisch sowie Fixation mittels TFNA© (130°, 170 mm Länge, 12 mm Durchmesser), bei leicht distaler und dorsaler Positionierung der Schenkelhalsklinge in den a.-p. bzw. axialen Bildverstärker(BV)-Aufnahmen (Abb. 2). Die Schenkelhals-Klingenspitze zeigte eine „tip apex distance“ (TAD) [8, 10] von 2–3 cm in den beiden BV-Ebenen. Das Einschlagen der Klinge erfolgte nach Aufbohren der lateralen Kortikalis mit dem entsprechenden Spiralbohrer, anschließend mit dem 2‑Stufen-Bohrer bis auf 85 mm, d. h. 10 mm kürzer als die gewählte 95 mm lange Spiralklinge. Der Führungsdraht lockerte sich nicht heraus; es fand keine Perforation ins Hüftgelenk statt. Anschließend erfolgten proximal die dynamische sowie distal die statische Verriegelung sowie der schichtweise Wundverschluss.

**Figure 3:**
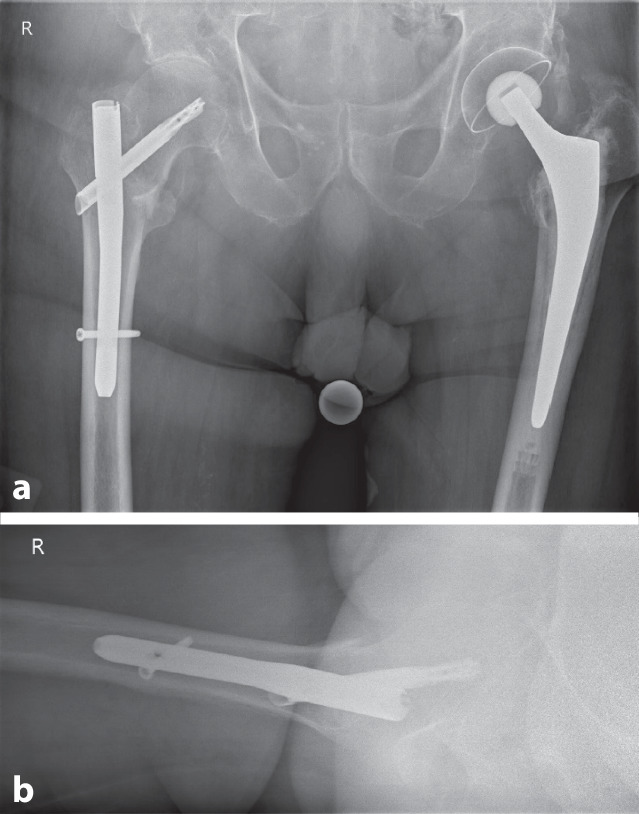

Herr A. wurde unter Vollbelastung mobilisiert. Die postoperative Röntgenkontrolle zeigte eine adäquate Knochen- und Implantatstellung, bei typischer Lateralisierung der Spiralklinge infolge einer Frakturkompression im Vergleich zu den BV-Bildern (Abb. 3). Verlegung am 8. postop. Tag in die Rehabilitation und nach komplikationslosem Verlauf am 34. postop. Tag Entlassung des Patienten nach Hause.

**Figure 4:**
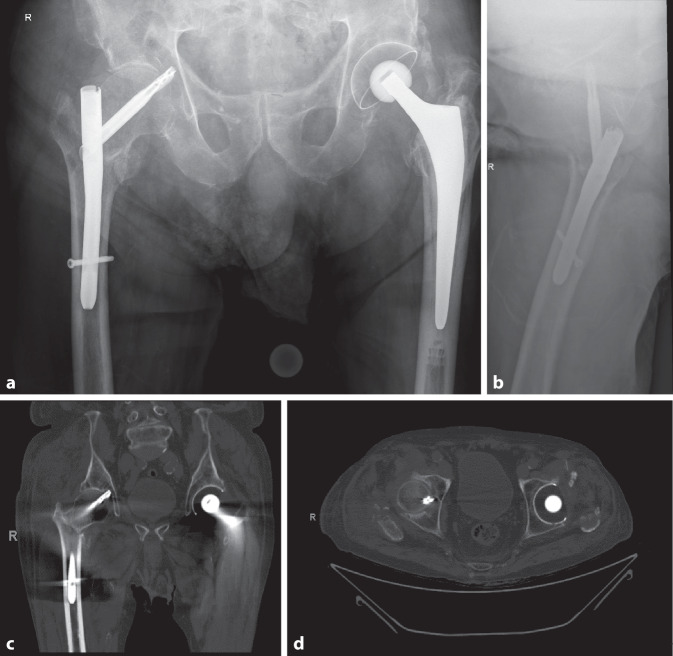

**Figure 5:**
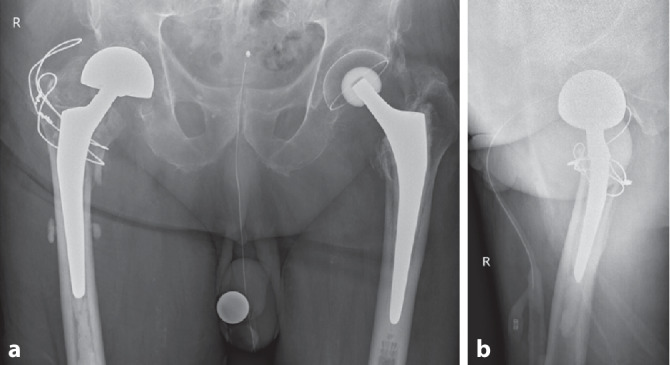

Aufgrund zunehmender Hüftschmerzen rechts wies ihn sein Hausarzt am 41. postop. Tag auf die Notfallstation ein. In der Röntgenkontrolle zeigte sich ein „cut-in“, d. h. eine „Wanderung“ der Schenkelhalsklinge ins Hüftgelenk, sodass die Klinge lateral den Nagel nicht mehr überragte und die Fraktur neu etwas klaffte bzw. sich varisierte. In der zusätzlich durchgeführten Computertomographie des Beckens zeigte sich kein Hinweis für eine Auslockerung der Klinge oder einen relevanten Acetabulumschaden (Abb. 4), bei Penetration der Hüftklinge gen Fossa acetabuli. Nach Abwägen der jeweiligen Vor- und Nachteile entschieden wir uns bei dem über 90-jährigen, vollantikoagulierten Patienten zum alleinigen Klingenwechsel und nicht zu einer Reosteosynthese oder einer prothetischen Versorgungmit dem Ziel eines möglichst kleinen Sanierungseingriffes. Dies auch unter der Annahme, dass gemäß Röntgen bzw. CT keine wesentliche Verkürzung mehr erfolgen würde, da die Frakturzonensinterung infolge „Anschlag“ des Femurkopf-Schenkelhals-Fragments lateral bzw. am Femurnagel bereits abgeschlossen sei. Diese Annahme erwies sich allerdings als falsch: Bereits frühpostoperativ zeigte sich unter erfolgter Vollbelastung eine weitere Sinterung, verbunden mit einem erneuten Cut-in der Klinge ins Gelenk, sodass eine Woche nach dem Klingenwechsel eine vollständige Metallentfernung mit endoprothetischer Versorgung durchgeführt wurde (Abb. 5). Nach kurzzeitigem Intensivaufenthalt postoperativ, bei u. a. akuter Niereninsuffizienz und Delirentwicklung, zeigte sich grundsätzlich ein komplikationsloser weiterer Verlauf. Bei der ambulanten Verlaufskontrolle knapp 3 Monate nach dem letzten Eingriff lief der wieder zu Hause mit Unterstützung selbstständig lebende Herr A. fast hinkfrei am Rollator, mit allein gelegentlicher Schmerzmitteleinnahme in Reserve. Es fand sich eine aktive Hüftbeweglichkeit mit Innen‑/Außenrotation 10/0/30° (Gegenseite 20/0/20°) und einer Hüftflexion von 110°. Er konnte im Liegen beide Beine von der Untersuchungsliege heben, das rechte Bein etwas schwächer, mit gut möglichen Ab- bzw. Adduktionsbewegungen, wenn auch reduziert in der Haltekraft des betroffenen Beines. Bezüglich der Osteoporosetherapie wurde gemäß Komplexbehandlungsschema auf unserer interdisziplinären alterstraumatologischen Abteilung eine Substitution mit Kalzium und Vitamin D per os durchgeführt. Zudem empfahlen wir eine Osteoporosebehandlung Typ Zoledronat einmal/Jahr als i.v.-Kurzinfusion oder Denosumab 2‑mal/Jahr s.c.

## Diskussion

Unser Fallbericht beschreibt unseres Wissens erstmals in der Literatur nach korrekter Implantation einen *vollständigen Cut-in* einer Schenkelhalsklinge nach TFNA-Versorgung einer trochantären Femurfraktur, d. h. die Medialisierung der gesamten Schenkelhalsklinge (auch im Nagel selbst) und nicht nur des Klingenanteils im Femurkopf-Hals-Fragment bei Fraktursinterung. Da das äußere Spiralklingenende sich initial lateralisierte (s. Vergleich der postoperativen Röntgen- zu den BV-Aufnahmen) und sodann als Ganzes gen Hüftgelenk „wanderte“, kann dieser Effekt nicht einer versehentlich statischen Verriegelung proximal zugeschrieben werden.

**Figure 6:**
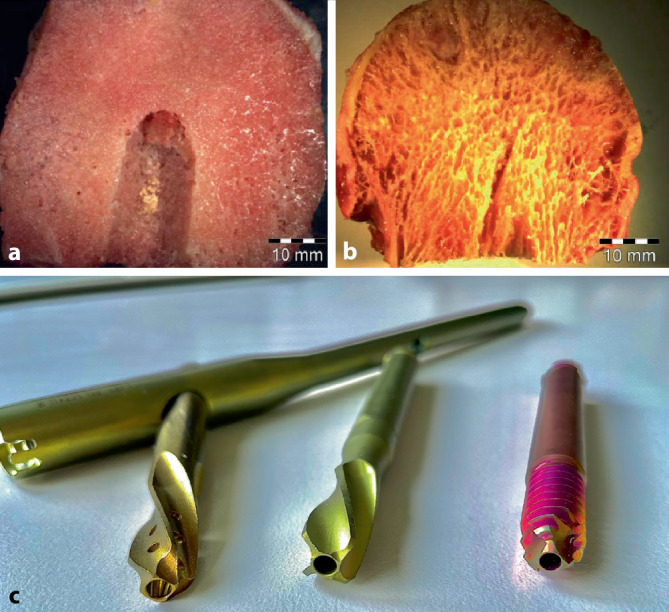

Unser Fall erscheint als Extrembeispiel für in den letzten Jahren zunehmende, zumindest partielle Cut-in-Ereignisse bei Spiralklingenimplantaten [11, 12]. Dieses Phänomen wurde z. B. für den PFNA erstmals 2012 von Frei et al. beschrieben [1]. In unserer eigenen Erfahrung hatten wir solche Fälle bei Schenkelhalsschraubenimplantaten praktisch nicht, aber auch beim Vorgängerimplantat PFNA (mit etwas stumpferer bzw. breiterer Klingenkonfiguration vs. dem TFNA; Abb. 6) seltener gesehen. Neuere Literaturangaben belegen eine deutlich höhere Cut-in-Rate der Klingen- vs. den Schraubenimplantaten [2, 5], allerdings gilt es, angesichts der zunehmend häufigeren Osteoporose bzw. älterer operierter Patienten derartige Kofaktoren mitzuberücksichtigen. Typischerweise hinkt die Fachliteratur bezüglich klinischer Evaluation neuer Implantate hinterher, was sich auch bezüglich PFNA und dessen Nachfolgeversion TFNA zeigt [11].

Die Anwendung des 1954 patentierten „Pohl-Laschenschrauben-Prinzips“, welches durch ihren zweiteiligen Gleitmechanismus das Risiko einer Gelenkperforation infolge unvermeidbarer Frakturkompression bei Gehbelastung nach Fixation einer pertrochantären Femurfraktur, z. B. mittels einer einteiligen Winkel- bzw. Klingenplatte, minimierte, war ein medizinhistorischer Meilenstein [10]. Mehrere Generationen unterschiedlichster Platten- und Nagelsysteme verschiedener Firmen verwenden dieses Prinzip bis heute modifiziert fort, wie z. B. die DHS, den PFNA, InterTAN, Gammanagel oder TFNA [2, 10, 11]. Mit deren Anwendung wird letztlich eine infolge der Fraktursinterung erfolgende „Lateralisierung“ der Femurkopf-Schenkelhals-Komponente als kleineres Übel im Vergleich zur „Medialisierung“ gen Acetabulum, mit dem Risiko der Hüftgelenkzerstörung, in Kauf genommen. Für den osteoporotischen Knochen wurden die bessere Knochenhaltekraft der propellerartigen Klinge bei verdrängend komprimierender umgebender Knochensubstanz vs. einzudrehender Hüftschraube nach Aufbohren gezeigt [4]. Allerdings lässt die Schärfe der Klinge im Gegenzug auch eine höhere Perforationsgefahr möglich erscheinen (Abb. 6). Trotz unterschiedlicher Implantatefortentwicklungen persistieren allerdings bei der osteosynthetischen Versorgung trochantärer Femurfrakturen nicht zuletzt geriatrischer Patienten typische Komplikationen in der Anwendung zephalomedullärer Implantate [10]. Dazu gehören mit einer Osteosyntheseversagensrate von 9–15 % gemäß Literatur neben der früher eher seltenen medialen Migration des Schenkelhalsimplantatanteils beim Cut-in (der eindeutigeren Verständlichkeit halber ziehen wir den Begriff Cut-in ansonsten in der Literatur benutzten Bezeichnungen wie „cut through“ oder „medial cut-out“ vor), der sog. Cut-out, d. h. das kraniale Ausschneiden der Schenkelhalsimplantatkomponente aus dem Femurkopf-Hals-Fragment [5]. Die beiden Komplikationsvarianten Cut-in und Cut-outwerden leider oftmals nicht voneinander getrennt, auch wenn diesen im Wesentlichen – abgesehen von der für beides mitverantwortlich gemachten Osteoporose – unterschiedliche Hauptursachen zugrunde liegen. Dies gilt es, in der täglichen Anwendung und zur Problemvermeidung zu verstehen: Für den Cut-out ist bei genauer Analyse meist eine ungenügende Frakturreposition oder Implantatpositionierung verantwortlich. Hingegen ist die Erklärungslage beim Cut-in abgesehen von z. B. irrtümlicher statischer Verriegelung des Gleitmechanismus des Schenkelhalsimplantatanteils (bei Implantaten wie dem Gammanagel oder TFNA), einer Blockade der Lateralisierung durch den „Z-Effekt“ (Scherwanderung bei 2 vorhandenen Schenkelhalsschrauben, z. B. beim PFN) [10] oder dem lateralen Anstehen zu weit in den Nagel eingebrachter Schenkelhalsschrauben oder -klingen am Nagelperforationsloch – nicht so klar. Vor allem bei herabgesetzter Knochenqualität (Osteoporose) scheint dies eine Problematik der scharfen, einzuschlagenden Spiralklinge gegenüber der stumpferen, einzudrehenden Hüftschraube zu sein. Seitens der Herstellerfirma des TFNA wird das Aufbohren im Hüftkopf-Hals-Fragment beim harten Knochen oder bei Benutzung der Schenkelhalsschraube empfohlen (https://www.jnjmedicaldevices.com/en-US/procedure/femur-fracture). Da das Vorliegen einer Osteoporose zum Operationszeitpunkt meist unsicher ist und wir Frakturdistraktionen ohne entsprechendes Aufbohren erlebt haben, verzichten wir bisher nicht standardmäßig auf den Stufenbohrereinsatz, limitieren diesen aber – wie im vorgestellten Fall – auf eine kürzere Strecke als die gewählte Klingenlänge. In der Literatur wird neben dem Verzicht auf das Aufbohren die sorgfältige Wahl des standardmäßig verwendeten Schenkelhalsimplantates empfohlen [1, 5]. Zukünftige Untersuchungen müssen zeigen, inwieweit je u. a. das Vorbohren bzw. dessen Durchmesser und Ausdehnung vs. Klingenkonfiguration und Einschlagmechanismus je nach Knochenqualität eine zusätzliche Perforationsgefahr mit sich bringen. Eine Zementaugmentation scheint gemäß neuerer Übersichtsarbeiten die Rate derartiger Fixationsversagen zu vermindern [3, 9], v. a. in Fällen mit dem Risiko eines hohen mechanischen Fixationsversagens und bezogen auf einen Cut-out. Dies ist im klinischen Alltag allerdings nicht immer erkenn- bzw. absehbar, insbesondere auch in unserem Falle eines Cut-in bei wohl grundsätzlich als osteoporotisch einzustufender Knochenqualität, aber repositions- bzw. implantationsmäßig adäquat erscheinender Frakturversorgung. Angesichts der Zementierungskosten bzw. der bisherigen Nichterstattung im DRG-System und der potenziellen Risiken ist somit aktuell noch nicht klar, welche Fallkonstellationen prospektiv gesehen effektiv von einer Augmentierung profitieren. Eine primäre endoprothetische Versorgung anstelle der initialen osteosynthetischen Versorgung ist für die pertrochantären Frakturen aufgrund der regelhaft diffizileren Versorgung bzw. schlechteren Resultate in den aktuellen Leitlinien nicht routinehaft vorgesehen, sondern bleibt Einzelfällen überlassen [6, 7, 10].

## Fazit für die Praxis

- Angesichts zunehmender Fälle sollte die Genese des Cut-in-Auftretens rasch detaillierter geklärt werden.
- Zwischenzeitlich sollten pragmatisch bei der osteosynthetischen Versorgung pertrochantärer Femurfrakturen geriatrischer Patienten v. a. im Falle der Anwendung von Spiralklingenimplantaten zurückhaltend aufgebohrt und gemäß aktueller Literatur eine Zementaugmentation eher großzügig erwogen werden.
